# Thin Films of Nanocrystalline Fe(pz)[Pt(CN)_4_] Deposited by Resonant Matrix-Assisted Pulsed Laser Evaporation †

**DOI:** 10.3390/ma14237135

**Published:** 2021-11-24

**Authors:** Dominik Maskowicz, Rafał Jendrzejewski, Wioletta Kopeć, Maria Gazda, Jakub Karczewski, Paweł Niedziałkowski, Armin Kleibert, Carlos A. F. Vaz, Yann Garcia, Mirosław Sawczak

**Affiliations:** 1 Photophysics Department, The Szewalski Institute of Fluid-Flow Machinery, Polish Academy of Sciences, Fiszera 14, 80-231 Gdańsk, Poland; rafj@imp.gda.pl (R.J.); wkopec@imp.gda.pl (W.K.); mireks@imp.gda.pl (M.S.); 2 Faculty of Applied Physics and Mathematics, Gdańsk University of Technology, Narutowicza 11/12, 80-233 Gdańsk, Poland; margazda@pg.edu.pl (M.G.); jakkarcz@pg.edu.pl (J.K.); 3 Faculty of Chemistry, University of Gdańsk, Bażyńskiego 8, 80-309 Gdańsk, Poland; pawel.niedzialkowski@ug.edu.pl; 4 Swiss Light Source, Paul Scherrer Institut, 5232 Villigen PSI, Switzerland; armin.kleibert@psi.ch (A.K.); carlos.vaz@psi.ch (C.A.F.V.); 5 Institute of Condensed Matter and Nanosciences, Molecular Chemistry, Materials and Catalysis (IMCN), Université Catholique de Louvain, Place Louis Pasteur 1, 1348 Louvain-la-Neuve, Belgium; yann.garcia@uclouvain.be

**Keywords:** temperature-dependent spin crossover, matrix-assisted pulsed laser evaporation, Fe(pz)[Pt(CN)_4_], resonant pulsed laser ablation, materials characterization, SOXIESST effect

## Abstract

Prior studies of the thin film deposition of the metal-organic compound of Fe(pz)Pt[CN]_4_ (pz = pyrazine) using the matrix-assisted pulsed laser evaporation (MAPLE) method, provided evidence for laser-induced decomposition of the molecular structure resulting in a significant downshift of the spin transition temperature. In this work we report new results obtained with a tunable pulsed laser, adjusted to water resonance absorption band with a maximum at 3080 nm, instead of 1064 nm laser, to overcome limitations related to laser–target interactions. Using this approach, we obtain uniform and functional thin films of Fe(pz)Pt[CN]_4_ nanoparticles with an average thickness of 135 nm on Si and/or glass substrates. X-ray diffraction measurements show the crystalline structure of the film identical to that of the reference material. The temperature-dependent Raman spectroscopy indicates the spin transition in the temperature range of 275 to 290 K with 15 ± 3 K hysteresis. This result is confirmed by UV-Vis spectroscopy revealing an absorption band shift from 492 to 550 nm related to metal-to-ligand-charge-transfer (MLCT) for high and low spin states, respectively. Spin crossover is also observed with X-ray absorption spectroscopy, but due to soft X-ray-induced excited spin state trapping (SOXIESST) the transition is not complete and shifted towards lower temperatures.

## 1. Introduction

Spin crossover (SCO) materials are widely investigated due to their ability to switch their spin state upon the influence of external conditions, such as temperature [1], light irradiation [2], and pressure [3]. Various proposed applications, such as in electronics, memory devices, molecular switches, or sensors [4], require nanoscale size and functionality at room temperature. Nanomaterials based on metal-organic frameworks (MOF) such as Fe(pz)[Pt(CN)_4_] (pz = pyrazine) are among the most promising ones due to the spin crossover effect and large hysteresis at or near this temperature. A great effort has been made to obtain functional thin films of SCO compounds using various deposition techniques (Langmuir–Blodgett [5], spin coating [6], dip coating [7], sequential assembly [8], etc.). However, MOFs are in general problematic to process due to the fact that their spin-crossover properties are highly dependent on crystallinity, the film’s 3D structure, and molecular environment [9,10,11]. Recently, we have proposed the application of the matrix-assisted pulsed laser evaporation (MAPLE) technique as a one-chamber method to obtain uniform and functional thin films [12]. The MAPLE technique is a derivative of the pulsed laser deposition (PLD) but, instead of direct material ablation, a cryogenically frozen suspension of deposited nanocrystalline material fulfills the role of the target transferred onto suitable substrates as a result of pulsed laser-induced evaporation. The solvent used in this process, also called a matrix, serves as a protector (absorbing the majority of laser energy) and as a carrier (during ablation, dispersed nanocrystals are carried with the matrix droplets towards substrate) [13,14,15]. The first of these roles is the major advantage of the MAPLE method as it prevents significant damage of SCO nanocrystals, which are highly susceptible to chemical decomposition, structural collapse, and deterioration of the spin-crossover properties.

An important factor for the MAPLE process is to select the appropriate laser wavelength so that it coincides with the maximum absorption of the matrix. In our previous reports [12,16,17,18], we presented the results obtained using 1064 nm neodymium-doped yttrium aluminum garnet (Nd:YAG) laser radiation. Although the radiation at this wavelength is relatively poorly absorbed by the matrix, studies have shown that for certain materials it is possible to obtain fully functional thin films of the nanocrystalline material [16]. However, in the case of metal-organic frameworks such as Fe(pz)[Pt(CN)_4_], the spin-crossover properties differed from reference nanocrystals, which was attributed to a not fully understood mechanism of material alteration under laser radiation [17]. Possible reasons associated with the combination of laser irradiation and vacuum influence as causing a lock of the high spin (HS) state upon pyrazine absorption, were discussed in another publication [18].

This paper presents a novel approach to the deposition of uniform and functional thin films of the Fe(pz)[Pt(CN)_4_]. We applied a new experimental setup equipped with a tunable pulsed laser with an optical parametric oscillator (OPO) in the infrared range, which allows us to match the laser wavelength to the vibrational bands of the solvent used as a matrix. Specifically, the mid-infrared wavelength, matched to water O-H oscillation bands (~3 µm), is used to maximize the resonant laser energy absorption. According to data available in the literature [19], the absorption coefficient for water is orders of magnitude larger at 3080 nm than 1064 nm used in our previous works. The low laser impact on the dispersed nanocrystals results in a remarkable improvement in the quality of the thin film structure compared to the results obtained after processing with Nd:YAG laser of fundamental wavelength. 

## 2. Materials and Methods

### 2.1. Fe(pz)[Pt(CN)_4_] Nanocrystal Preparation

Fe(BF_4_)_2_·6H_2_O, K_2_Pt(CN)_4_·3H_2_O and pyrazine were purchased from Sigma-Aldrich (Saint Louis, MO, USA), sodium bis-(2-ethylhexyl) sulfosuccinate from Acros Organics (Thermo Fisher Scientific, Geel, Belgium), and n-octane from Alfa Aesar (Thermo Fisher Scientific, Kandel, Germany). All chemicals were used without additional purification. The synthesis of [Fe(pz)(Pt(CN)_4_]·2.5H_2_O was performed based on the procedure described by Boldog et al. [20] with some modifications, namely under constant temperature (20 °C) and under an inert atmosphere of reaction. Additionally, the obtained microemulsions were added gradually over a period of 30 min, while in the method described by Boldog they were transferred quickly. These modifications were carried out in order to minimize the particle size dispersion. Initially, the mixture consisting of Fe(BF_4_)_2_·6H_2_O (67.4 mg, 0.2 mmol) and pyrazine (64 mg, 0.8 mmol) was dissolved in 2 mL of water and stirred under a nitrogen atmosphere. Then the obtained solution was added dropwise to the separate flask containing a mixture of sodium bis-(2-ethylhexyl) sulfosuccinate (9.87 g, 22.2 mmol) dissolved in 44 mL of n-octane. The mixture was stirred keeping 20 °C under an atmosphere of nitrogen to obtain the microemulsion. Then, in another flask, the K_2_Pt(CN)_4_·3H_2_O (86.2 g, 0.2 mmol) was dissolved in 2 mL of water and added dropwise to the solution containing sodium bis-(2-ethylhexyl) sulfosuccinate (9.87 g, 22.2 mmol) dissolved in 44 mL of n-octane to obtain the second microemulsion. The two microemulsions were stirred for 15 min. Then the solution of the first microemulsion was dropwise added to the flask with the second microemulsion for 30 min. The obtained final mixture was stirred at 20 °C under a nitrogen atmosphere for 48 h. The orange solid product was isolated by centrifugation (3000 rpm, 5 min), then washed four times with ethanol and dried at room temperature in air. The crude product was dried at 80 °C for 24 h to reduce the amount of structural water, to yield 65 mg of nanocrystalline powder.

### 2.2. Thin Film Preparation

The scheme of the MAPLE deposition setup was described in detail in our previous papers [16,17,18]. A suspension of SCO material was prepared by dispersing the nanocrystalline powder in deionized water to obtain 1 wt %. concentration. The suspension was sonicated for 10 min until a uniform color was obtained. Substrates (10 × 10 × 0.3 mm^3^ monocrystalline (100) Si plates and 10 × 10 × 0.15 mm^3^ glass plates for absorption UV-Vis measurements) were sonicated in acetone, rinsed using isopropanol, and dried. The substrates were mounted on a holder with a substrate-target distance set to 15 mm. The target holder was cooled using a helium cryostat (Leybold, London, UK) to 80 K at a backing pressure of 10^−5^ mbar. Next, the chamber was filled with dry nitrogen to prevent evaporation of the Fe(pz)[Pt(CN)_4_] suspension in a vacuum, and then the suspension was injected into a target holder reservoir. After target solidification, the chamber was evacuated again to backing pressure of 10^−5^ mbar. To achieve ablation, a tunable laser source with a 6 ns pulse duration (Radiant X 2731 + 3034, Opotek, Carlsbad, CA, USA) was set to 3080 nm. The target surface was scanned by the laser beam (10 Hz) with the energy of (9 ± 0.2) mJ, and laser spot size (0.5 ± 0.1) mm resulting in a fluence of (0.8 ± 0.1) J/cm^2^. The deposition time was set to 90 min. The reference samples were prepared by drop-casting of 50 µL of 1 wt %. suspension on an adequate substrate.

### 2.3. Structural Analysis

Surface imaging was carried on using a scanning electron microscope (SEM-FEG, FEI Quanta 250, Thermo Fisher Scientific, Hillsboro, OR, USA). X-ray diffraction (XRD) patterns were acquired using an X-ray diffractometer (X’Pert PRO MPD, PANalytical, Almelo, The Netherlands) equipped with a Cu-Kα (1.541 Å) X-ray source. Thin film surface profiles were analyzed with a DektatXT (Bruker, Billerica, MA, USA) profilometer with a 2.5 µm probe.

### 2.4. Spin-Crossover Analysis

SCO properties were examined by means of Raman, UV-Vis absorption, and X-ray absorption spectroscopy techniques. Raman spectra were obtained using a micro-Raman spectrometer (InVia, Renishaw, Wotton-under-Edge, UK) equipped with liquid nitrogen cryostats (MicrostatN, Oxford Instruments, Long Hanborough, UK) with automatic temperature control (Mercury ITC, Oxford Instruments, Long Hanborough, UK). As the excitation source, a 785 nm < 0.2 mW laser was used. Samples were analyzed over the temperature range from 150 to 320 K both on cooling and heating with 10 and 5 K (near the predicted spin switch temperature) intervals. Each Raman spectrum was averaged 10 times to improve the signal-to-noise ratio. Absorption spectra were acquired using a UV-Vis Lambda 35 spectrometer (Perkin Elmer, Waltham, MA, USA) in a transmission mode with a 1 nm scan interval and 120 nm/min speed. The absorption spectrometer was equipped with a custom build cryostat unit controlled with a Mercury ITC temperature controller. The temperature was changed in the same range as in the Raman measurements. To prevent water condensation on the samples during the measurements in the low-temperature range, the cryostat interior was purged with dry nitrogen at normal pressure.

X-ray absorption spectroscopy (XAS) was carried at the Surface and Interface Microscopy (SIM) beamline of the Swiss Light Source (Paul Scherrer Institut, Switzerland). The samples were placed in a cold finger of a variable temperature cryostat and measured with linearly polarized X-ray light incident perpendicular to the film surface. The XAS signal was measured in both total electron yield (TEY), by measuring the electron current that compensates the photoemitted electrons and secondaries, and the total fluorescence yield (TFY) using a photodiode placed next to the sample. The two acquisition methods provide different probing depths, of a few nm for TEY and hundreds of nm for TFY.

## 3. Results and Discussion

### 3.1. Structural Characterization

**Figure 1:**
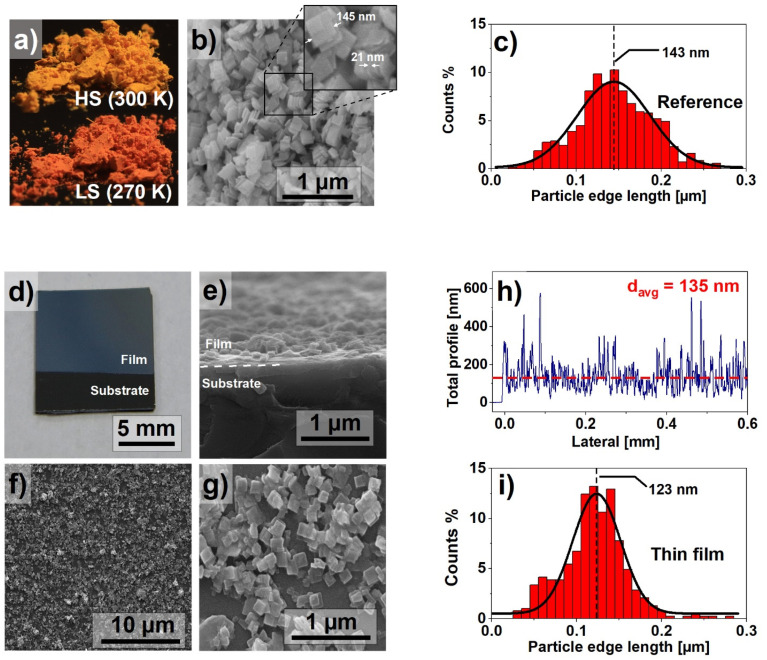
(**a**) Fe(pz)[Pt(CN)_4_] in the high spin (HS) and low spin (LS) states; (**b**) scanning electron microscope image of nanocrystals; (**c**) particle size histogram based on the dimensions calculated from SEM images; (**d**) macroscopic photograph of the prepared thin film; (**e**) SEM cross-section of the thin film; (**f**,**g**) SEM images of the surface of the thin film; (**h**) results of profilometry measurement performed on the thin film; (**i**) particle size histogram of the thin film particle size distribution.

Since the properties of the SCO materials, such as the spin switching temperature or the width of the hysteresis loop, are determined, among other factors, by the size of the nanocrystals [21], an important goal of the experiment was the synthesis of nanocrystals with the smallest possible size dispersion, described in Section 2. Figure 1a shows images of the materials as synthesized. The powder, bright yellow at room temperature, turn red when cooled below 270 K. Figure 1b presents the SEM image of the powder. The nanocrystals are in the form of flat, square plates with an average side length x ≈ 145 nm and a thickness d ≈ 21 nm. The nanocrystals size dispersion was determined on the basis of the analysis of the SEM images. It confirmed the small dispersion of the crystallite size. The size distribution is presented as a histogram in Figure 1c. The results indicate a near Gaussian size distribution with the majority of the nanocrystals edge lengths varying between 100 and 200 nm. Such distinction between x and d dimensions directly affects the morphology of prepared thin films.

The macroscopic photograph of the obtained thin film is shown in Figure 1d, suggesting a uniform filling of the surface. The detailed analysis by the SEM imaging of the MAPLE-deposited film shows planar-oriented nanocrystallites on the surface of the silicon substrate. Both cross-section (Figure 1e) and top-view (Figure 1f,g) scans indicate fair homogeneity; however, a small number of agglomerates can be observed. These agglomerates are the result of clustering of the nanocrystals during synthesis, but also of random particle distribution during the deposition process. The presence of clusters is difficult to avoid, but their small number does not appear to have a significant impact on both the quality and properties of the film. Despite the random distribution of the nanocrystals, the surface filling of the obtained samples is sufficiently good. The described thin film morphology was also confirmed using profilometry. A representative result is presented in Figure 1h. The average film thickness was estimated as 135 ± 20 nm. The peaks observed in the profilometry graph are caused by vertically stacked nanocrystals. Similar to the results presented in Figure 1c, Figure 1i presents a particle size histogram for the thin film. The narrower particle size distribution in the case of thin films can be explained by selective deposition of nanocrystals, dependent on their kinetic energy, as reported in our previous paper [17].

**Figure 2:**
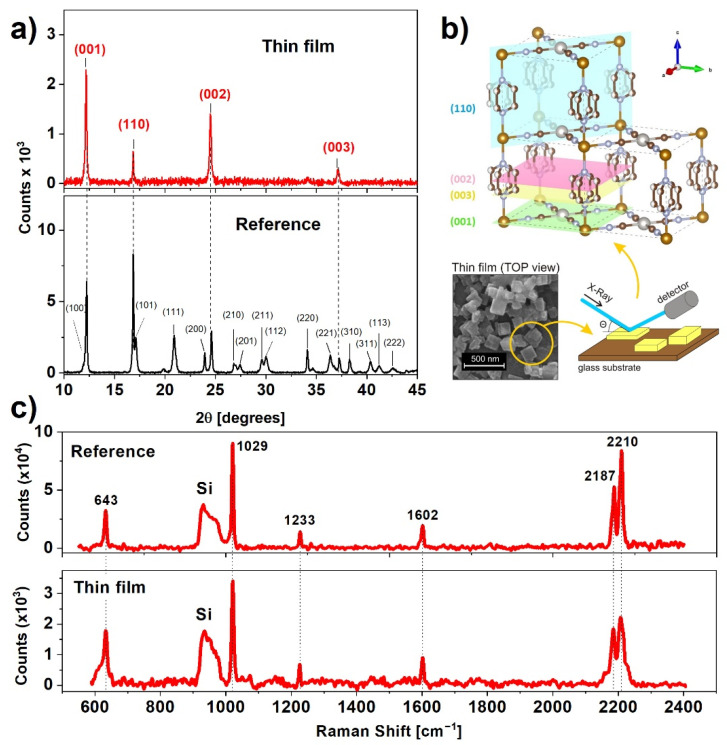
(**a**) X-ray diffraction pattern of the reference and thin film samples indexed to the respective Miller planes; (**b**) position of major Miller planes of Fe(pz)[Pt(CN)_4_] and visualization of the effect of texture on the diffraction patterns; (**c**) comparison of wide range Raman spectra of the reference sample and thin film.

In order to examine the crystalline structure of the prepared samples, we carried out X-ray diffraction (XRD) measurements (Figure 2a). The XRD pattern of the reference sample is nearly identical to XRD to that presented by Alvarado et al. [22]. The positions of the reflections indicate tetragonal crystalline structure with the following unit cell parameters: *a* = *b* = 7.45 Å, *c* = 7.26 Å, which are congruent with the results for the high spin state of non-dehydrated nanocrystals [23]. According to Ohba et al. [24], the water content of this complex can be estimated to be 1–2 molecules per unit cell.

The XRD pattern of the thin film indicates four reflections from planes shown in Figure 2b, generated using the VESTA (version 3.5.7) software, namely (001), (110), (002), and (003), indicating a preferential alignment of the nanocrystals along the [001] direction [25]. This result, in combination with the SEM micrographs (Figure 1f,g), shows that the plate-like structure of nanocrystals favors an arrangement with a larger surface plane facing the substrate. During synthesis, the crystallization process favors a direction parallel to Pt-CN-Fe (directed as presented in Figure 2b), which was confirmed by Delgado et al. [21]. The positions of the diffraction peaks for the film are nearly the same as for the reference sample, indicating that the water content remained unchanged after the deposition process. The unchanged structure was also confirmed by a comparison of Raman spectra for the reference sample and thin film. Characteristic Raman bands centered at 643, 1029, 1233, 1602, 2187, and 2210 cm^−1^ are exactly the same for both samples and agree with values presented in the literature [26].

Hence, the XRD and Raman results confirm that the 3080 nm laser irradiation during MAPLE deposition does not cause any significant structural damage to the deposited nanocrystals. As predicted, the water matrix fulfills its function as a laser radiation absorber and protector for the fragile nanocrystalline framework of Fe(pz)[Pt(CN)_4_].

### 3.2. Spin-Crossover Properties

#### 3.2.1. Temperature-Dependent Raman Measurements

**Figure 3:**
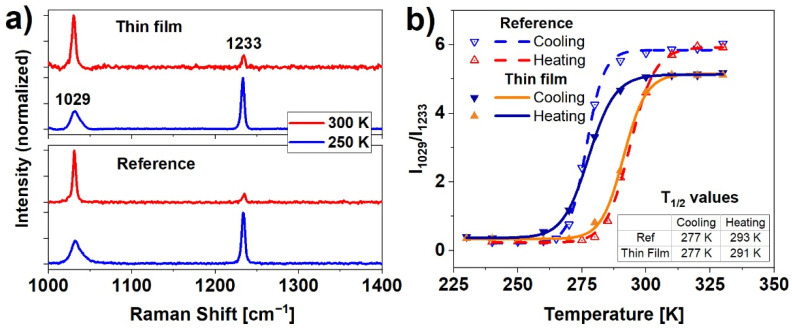
(**a**) Comparison of HS (300 K) and LS (250 K) Raman spectra of the reference sample and thin film; (**b**) hysteresis of the ratio of the intensities of the 1029 to 1233 cm^−1^ bands for the reference sample and the thin film obtained from temperature-dependent Raman measurements.

The Raman spectra were used to determine spin switching evolution as a function of temperature. The spin transition in Fe(pz)[Pt(CN)_4_] is caused by a change in the number of electrons in antibonding and nonbonding orbitals. Differences in HS (t_2g_^4^e_g_^2^) and LS (t_2g_^6^) configurations translate into a change of metal-ligand bond length, and further, to a deformation of the shape of the crystal framework [27]. In this particular case, to detect SCO upon nanocrystalline material, two distinct bands were examined: 1029 cm^−1^, which corresponds to pyrazine ring stretching vibrations, and 1233 cm^−1^ related to CH ligand in-plane bending [26]. In both reference and thin film samples (Figure 3a) the Raman spectra show these characteristic strong vibration modes. The analysis of the ratio of the intensities of the 1029 cm^−1^ to 1233 cm^−1^ bands as a function of temperature reveals hysteretic dependence of the spin state population, presented in Figure 3b. The spin transition temperatures (commonly described as T_1/2_) were calculated by determining the inflection points of logistic curves fitted to the measured data points. Values of T_1/2_ (calculated as the average of five measurements) equals for cooling and heating respectively: T_1/2_^↓^ = 277 K, T_1/2_^↑^ = 293 K for the reference sample, and T_1/2_^↓^ = 277 K and T_1/2_^↑^ = 291 K for thin film. The hysteresis width for the reference sample (16 ± 3 K) and the thin film (14 ± 3 K) indicate a remarkable thermally induced SCO effect indicating strong and consistent nanocrystals cooperativity. The slight difference in the hysteresis between film and reference sample can be a sum of a few factors such as small differences in the heat conduction (a consequence of the different thickness of these samples) or a slightly different amount of water contained in the nanocrystalline structure.

#### 3.2.2. Temperature-Dependent UV-Vis Absorption Measurements

**Figure 4:**
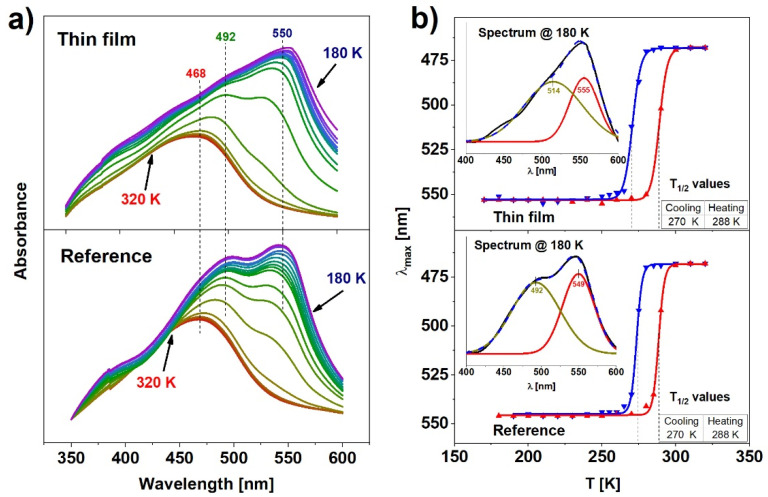
(**a**) Evolution of absorption spectra for reference and thin film; (**b**) hysteresis obtained from absorption spectra for both samples.

Complementary to the Raman analysis, temperature-dependent UV-Vis transmission absorption spectroscopy measurements were performed for both reference and thin films samples. Although the spectra were recorded for cooling and heating cycles, they do not differ visibly (except for the shift in temperature related to the hysteresis), therefore the description will focus on the representative cooling cycle shown in Figure 4a. All spectra were corrected with baseline subtraction determined as a ~λ^−4^ scattering dependence. The absorbance spectra of both samples in the high spin state at 320 K exhibit a single band located at 468 nm. As the temperature is reduced and the number of molecules in the low spin state grows, the band shifts towards longer wavelengths and its intensity raises significantly for both samples. Simultaneously, during cooling, an additional band centered near 550 nm appears. After the deconvolution of the spectra obtained at 180 K, two separated Gaussian absorption bands can be distinguished (Figure 4b, inset). The band centered at 492 nm in the case of the reference sample is shifted to 514 nm for the thin film, whereas the band centered at 549 nm for the reference sample is shifted by 6 nm towards the longer wavelengths. The band centered near 492 nm could be attributed to the metal-to-ligand-charge-transfer (MLCT) [28]. MLCT may occur when the transition metal is in a low electronic state and the ligand possesses low-lying empty orbitals. Then, the excitation of an electron from the metal d orbital to the antibonding π* level of the ligand is possible [29]. As the sample switches to the LS state, the electrons leave the antibonding d orbitals. This creates the possibility of a transition within the d level. The d-d electronic band emerges on the right-hand side of the MLCT (549 nm for the reference sample, 555 nm for the thin film) because the energy difference between d orbitals is smaller than the energy required for the MLCT transfer.

The above-mentioned absorption band shift, corresponding to the HS → LS transition, agrees with the color (yellow → red) change at the spin transition. The quantitative changes of the LS and HS populations can be estimated from the positions of the maxima of the absorption spectra. Figure 4b shows the temperature dependence of these changes in spectra in the thin film (top) and the reference sample (bottom). Values of T_1/2_ determined from these results are almost identical in both cases and result in 15 ± 3 K wide hysteresis loops. Such outcome indicates that the thin film of Fe(pz)[Pt(CN)_4_] exhibits cooperative properties as well as the reference sample and can be bistable under a relatively wide range of temperatures. The results obtained from the UV-Vis analysis are in general agreement with the Raman spectroscopy results, thus it can be concluded that these methods allow one to determine the spin state of the material unambiguously.

#### 3.2.3. X-ray Absorption Measurements

**Figure 5:**
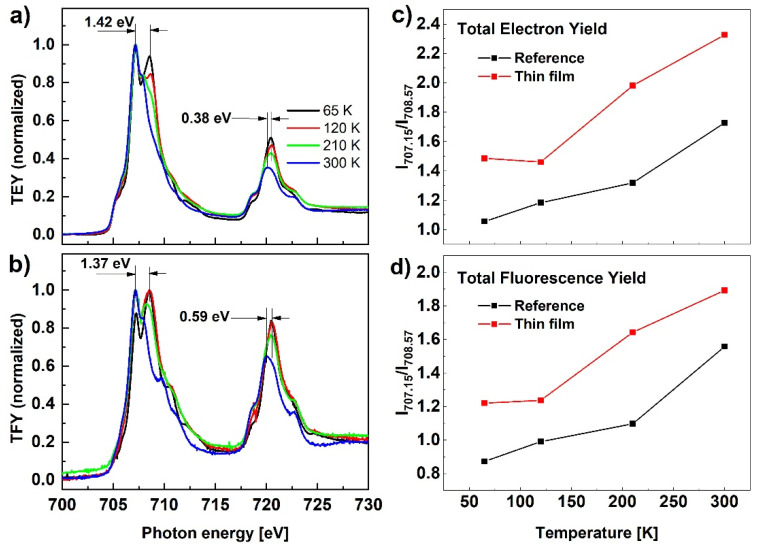
(**a**,**b**) XAS in TEY and TFY detection modes for the reference sample of nanocrystalline Fe(pz)Pt[CN]_4_ recorded at different temperatures; (**c**,**d**) L_3_-edge relative peak intensity measured at 707.15 eV and 708.57 eV corresponding to peak positions for HS and LS, respectively.

The results of the X-ray absorption spectroscopy measurements at the Fe L_2,3_-edge for the Fe(pz)Pt[CN]_4_ reference sample are presented in Figure 5 at four temperatures, 65, 120, 210, and 300 K. The two main peaks at energies of around 710 eV and 723 eV correspond to the L_3_ and L_2_ edges, respectively, associated to transitions from the 2p core-level states to empty states in the 3d orbitals (where the energy difference between the L_3_ and L_2_ edges corresponds to the spin-orbit split 2p^1/2^ and 2p^3/2^ states). Compared to the spectrum at 300 K, the spectrum recorded at 65 K is shifted to higher energies: 1.42 eV for L_3_ and 0.38 eV for L_2_-edge maxima. According to results available in the literature, the observed transformation confirms the transition from the high spin to the low spin state [30,31], however, the results presented in Figure 5a show an incomplete spin transformation. This observation is not consistent with the spin transformation results confirmed with Raman or UV-Vis measurements and we assign it to the soft X-ray-induced excited spin state trapping (SOXIESST) effect. It has been reported that the HS state can be trapped under X-ray irradiation well below T_1/2_ [32,33]. From the XAS spectra shape evolution, it can be concluded that spin transformation takes place gradually as the temperature is reduced and is not complete even at the lowest temperature. This effect is more clearly visible in Figure 5c,d presenting the relative intensity evolution of the L_3_-edge measured at 707.15 eV and 708.57 eV, corresponding to the peak positions of HS and LS, respectively. Analogous spin transformation characteristics can be concluded from the TFY signal presented in Figure 5b, and the same follows for the spin evolution with temperature (Figure 5d) showing that the behavior at the sample surface is similar to the bulk of the nanoparticles.

**Figure 6:**
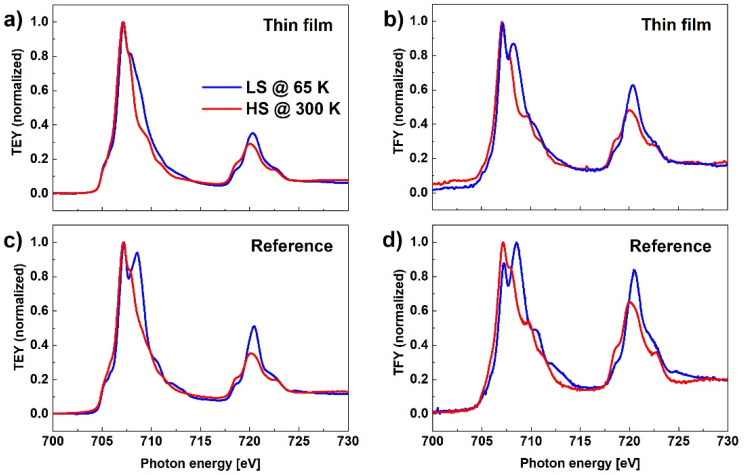
XAS in TEY (**a**,**c**) and TFY modes (**b**,**d**) for the reference and MAPLE-deposited samples measured at two temperatures, 300 K and 65 K.

Figure 6 shows XAS for both TEY and TFY detection modes and for both reference and MAPLE-deposited samples measured at two temperatures, 300 K and 65 K. Comparing the results obtained for the reference sample where the SCO layer thickness is in the range of a few tens of micrometers to the MAPLE-deposited sample, where the average thickness of the film is estimated as 135 nm, the relative intensity of the peak at 708.57 eV, characteristic for LS, to the peak at 707.15 eV, characteristic for HS, is significantly smaller in case of the thinner film. This result can be again related to the SOXIESST effect that is more evident in the case of the thinner film. Similar results, confirming that the SOXIESST phenomenon has less influence in the case of powder samples, have been reported by other authors [30]. Probably the SCO nanocrystals in the thick layer of the reference sample are sparsely distributed, so that the actual intensity of the X-ray beam incident on the SCO complex is weaker than the nominal value, while in the case of a MAPLE-deposited layer, the nanocrystals form a thin film on the substrate surface.

## 4. Conclusions

In summary, a resonant infrared MAPLE technique was applied for the fabrication of fully functional and homogenous thin films of nanocrystalline metalorganic SCO material. The nanocrystalline Fe(pz)[Pt(CN)_4_] powder used as SCO was synthesized according to an optimized chemical procedure to produce the material with a small crystal size distribution. This material was characterized using XRD, SEM, and Raman techniques. SEM analysis confirmed that the nanocrystals are in the form of plates with average dimensions of 143 × 143 × 21 nm^3^. When deposited as a thin film using MAPLE on the Si or glass substrates, the crystals had a distinct tendency to lay flat on the substrate. The orderly arrangement of the crystals on the surface was also confirmed by the XRD analysis, in which enhanced reflections from selected crystal planes were observed. The deposited films were homogenous, with only a small number of clusters of nanocrystals. The spin switching properties were investigated by means of three techniques: Raman spectroscopy, UV-Vis absorption spectroscopy, and X-ray absorption spectroscopy. Results of Raman and UV-Vis measurements, carried out as a function of temperature, showed comparable characteristics with the spin switching at temperatures near 277 K and 290 K while cooling and heating, respectively. The X-ray absorption spectroscopy measurements conducted for the reference material and thin film samples confirmed the spin crossover, however, due to the SOXIESST effect (which was more pronounced in the case of thin film samples), the spin conversion was not complete. The summary presented in Table 1 indicates a significant improvement of deposited films quality compared to the results reported in our previous papers.

In conclusion, the results presented here demonstrate that fragile metalorganic nanocrystalline SCO materials can be successfully deposited using a physical MAPLE technique capable of being easily scaled up and adapted to the production process.

## Figures and Tables

**Table 1:** Comparison of MAPLE deposition of Fe(pz)[Pt(CN)_4_] using 1064 [17,18] and 3080 nm lasers.

Parameter	1064 nm Laser	3080 nm Laser
Ambient pressure	Near atmospheric pressure required	Deposition in vacuum available
Matrix	Deionized H_2_O	Deionized H_2_O
Interaction laser–matrix	Weak, high laser fluence required to induce ablation (>2 J/cm^2^)	Strong, low laser fluence required (0.8 J/cm^2^)
Interaction laser–Fe(pz)[Pt(CN)_4_]	Possible decomposition due to poor matrix absorption	Negligible
Film quality	Poor uniformity with large clusters	Good uniformity; small, sparse clusters
SCO temperature range compared to the reference	Significant downshift	Unchanged (near room temperature)

## Data Availability

Data contained within the article is available from the corresponding author on request.
